# Treatment of severe osteochondral defects of the knee by combined autologous bone grafting and autologous chondrocyte implantation using fibrin gel

**DOI:** 10.1007/s00167-012-1891-z

**Published:** 2012-02-03

**Authors:** Yvonne E. Könst, Rob J. Benink, Ron Veldstra, Tjerk J. van der Krieke, Marco N. Helder, Barend J. van Royen

**Affiliations:** 1 Department of Orthopaedic Surgery, Gemini Ziekenhuis, Huisduinerweg 3, 1780 AT Den Helder, The Netherlands; 2 Department of Orthopaedic Surgery, VU University Medical Center, De Boelelaan 1117, 1081 HV Amsterdam, The Netherlands

**Keywords:** Osteochondral defect, Knee, Bone graft, Fibrin gel, ACI

## Abstract

**Purpose:**

Severe symptomatic and unstable osteochondral defects of the knee are difficult to treat. A variety of surgical techniques have been developed. However, the optimal surgical technique is still controversial. We present a novel technique in which autologous bone grafting is combined with gel-type autologous chondrocyte implantation (GACI).

**Methods:**

Isolated severe osteochondral defects of the medial or lateral femoral condyle were treated by a two-step procedure. Firstly, chondrocytes were harvested during arthroscopy and cultured for 6 weeks. Secondly, a full thickness corticospongious autologuos bone graft, harvested from the medial or lateral femur condyle, is impacted in the defect and covered by GACI. The fibrin gel fills up to the exact shape of the chondral lesion and polymerizes within 3 min after application.

**Results:**

From 2009 to 2011, 9 patients, median age 35 years (range 23–47), were treated by the combined autologous bone grafting and GACI technique. Median defect size was 7.1 cm^2^ (range 2.5–12.0), and median depth of the lesion was 0.9 cm (range 0.8–1.2). Median follow-up was 9 months (range 6–12 months). Six patients were available for 12-month follow-up. The mean IKDC score showed a 6-month improvement from 35 (SD ± 16) to 51 (SD ± 18) (*n* = 9; *p* = 0.01), and a 1-year improvement from 35 (SD ± 16) to 57 (SD ± 20) (*n* = 6; *p* = 0.03). The mean KOOS improved from 44 (SD ± 16) to 62 (SD ± 19) (*n* = 9; *p* = 0.07) at 6-month follow-up and from 44 (SD ± 16) to 65 (SD ± 24) (*n* = 6; *p* = 0.1) at 12-month follow-up. There was one failure that needed conversion to a unicompartmental knee arthroplasty.

**Conclusion:**

Combined autologous bone grafting and GACI may offer an alternative surgical option for severe and unstable osteochondral defects of the knee.

**Level of evidence:**

IV.

## Introduction

Osteochondral defects are severe localized intra-articular defects of the cartilage and subchondral bone, leading to partial or complete detachment of the fragment. The knee joint is the most affected anatomical site [20, 26, 32]. Although König first described this lesion in 1887, the aetiology has not been clarified up to now [4, 17, 32].

Severe symptomatic and unstable osteochondral defects are difficult to treat. A variety of surgical techniques has been developed; however, the optimal surgical technique is still controversial [13, 27]. Fragment removal without subsequent repair may be considered in some cases [1, 24, 36]. Nevertheless, current treatment modalities are more focused on restoration of the defect. Internal fixation of the unstable osteochondral defect with metal or resorbable screws is the first option of treatment [12, 22, 23]. When fixation is not amendable, autologous matrix-induced chondrogenesis with bone grafting [6] or mosaic osteochondral autograft transplantation is described to treat osteochondral defects [15, 16].

Currently, there is a growing interest in regenerative techniques, such as autologous chondrocyte implantation (ACI), in the management of articular lesions [7, 8, 21, 31, 34]. In ACI, a full thickness cartilage graft is harvested from a non-loadbearing site, and chondrocytes are isolated by extracellular matrix digestion and subsequently cultured for four to 6 weeks. Thereafter, the autologous chondrocytes are implanted in the cartilage defect by injection of the chondrocyte suspension under a sutured and sealed tibial periosteal (ACI-P) or a porcine collagen (ACI-C) patch. In severe osteochondral defects, however, both subchondral bone and articular cartilage are damaged. Reconstruction of both structures, bone and cartilage, is of paramount importance for optimal healing of the defect. Only few papers describe the treatment of osteochondral defects by combined bone grafting and ACI [5, 21, 25, 28]. Cancellous bone from the iliac crest or from the proximal-lateral part of the tibia is harvested and followed by the ACI-P or ACI-C procedure.

These ACI-P and ACI-C techniques, however, require securing of a periosteal membrane or collagen membrane to the normal cartilage edge by resorbable sutures and fibrin glue. Recently, the use of a gel-type ACI (GACI) has been introduced as an alternative ACI procedure for the treatment of focal articular cartilage defects in the knee that does not need any additional securing of the periosteal or collagen flap [10, 18, 19, 37]. The fibrin gel fills up to the exact shape of the chondral lesion and polymerizes within 3 min after application.

A novel technique for the treatment of severe symptomatic osteochondral defects of the knee by combined autologous bone grafting and ACI in fibrin gel (GACI) is presented. The objective of this study is to describe the technique and report our short-term results of the first patients treated by this technique.

## Materials and methods

From March 2009 until December 2010, 9 patients, 3 men and 6 women, were treated for severe symptomatic and unstable isolated osteochondral defects of the lateral or medial femoral condyle by combined autologous bone grafting and GACI. All patients signed written informed consent before treatment. The median age at operation was 35 years (range 23–47), and the median follow-up was 9 months (range 6–12). Inclusion criteria for treatment were a history of long-term pain and swelling of the knee after exercise not responding to conservative treatment. Five patients suffered from a post-traumatic osteochondral defect, and four patients suffered from a symptomatic osteochondritis dissecans lesion. Plain radiographs and MRI were used to localize the osteochondral defect. The severity of the defect was staged by arthroscopy according to the International Cartilage Repair Society (ICRS) criteria [9]. The size and depth of the defect were measured in mm and expressed in cm allowing one decimal. The osteochondral defects of the medial or lateral femoral condyle were treated by a two-step procedure. Firstly, chondrocytes were harvested by arthroscopy and cultured for 6 weeks. Secondly, a full thickness corticospongious autologuos bone graft, harvested from the medial or lateral femur condyle, was impacted in the defect and covered by GACI. Two orthopaedic surgeons performed the surgical procedure (RB and RV).

Patient outcome was analysed using the international knee documentation committee (IKDC) evaluation form [2] and the knee injury and osteoarthritis outcome score (KOOS) [11] preoperative and at 3, 6, and 12 months postoperative. The IKDC consisted of three domains: symptoms, sport activities and function. Total score was limited from 0 to 100 points representing the disability of the patient, in which high scores indicate minimal disability and low scores indicate high limitations on the three domains. The KOOS questionnaire consisted of five domains: symptoms and stiffness, pain, activities of daily living, function in sports and recreational activities, and quality of life. Scores were transformed to a scale of 0–100 points, with 0 representing extreme knee problems and 100 representing no knee problems. Scores of each domain were added up and divided by the number of domains. Early MRI evaluation was performed 3 months postoperatively.

### Statistical analysis

Statistical analysis was done using a Wilcoxon rank test, and calculations were made using SPSS version 18.0. Statistical significance was defined as *p* < 0.05.

### Surgical procedure

During the initial arthroscopic staging of the defect, a cartilage biopsy was taken from the margin of the femoral condyle to obtain chondrocytes for culture expansion. The culturing process of the chondrocytes and the subsequent GACI procedure (Chondron^™^) were performed as described previously [10, 18, 19, 37].

**Fig. 1:**
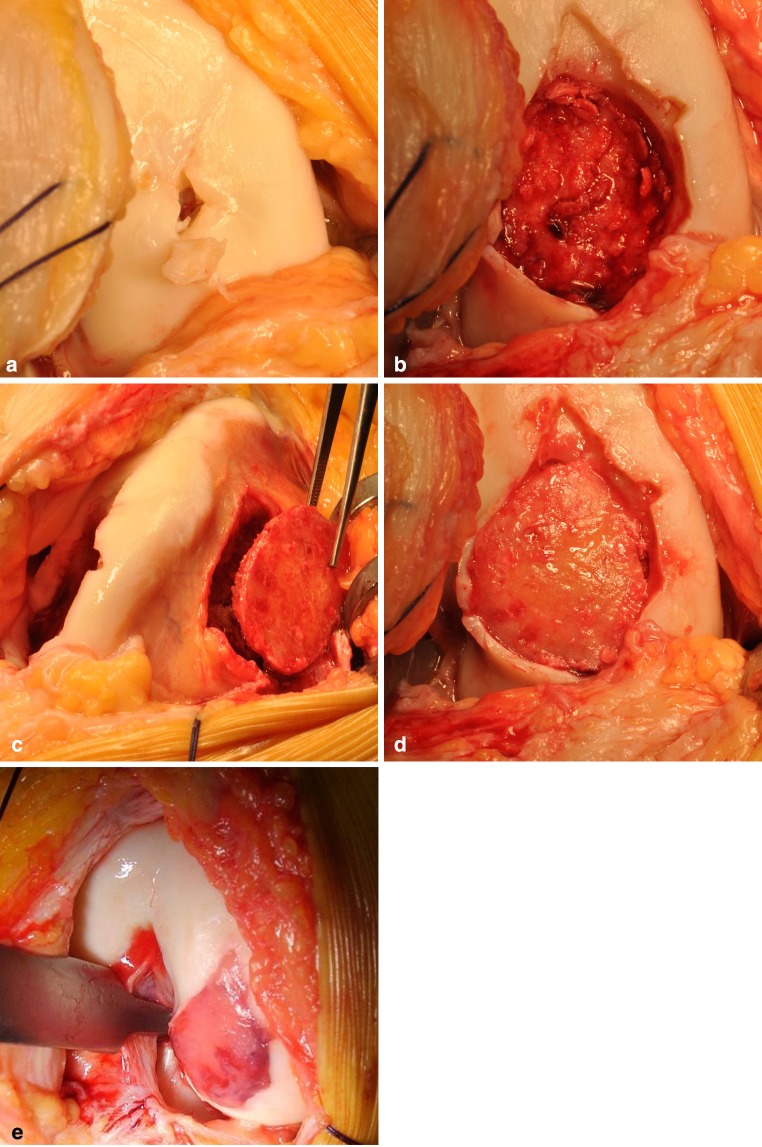
**a**–**e** Surgical procedure of combined autologous bone grafting and autologous chondrocyte implantation in fibrin gel in an unstable osteochondral defect of the knee: **a** preoperative macroscopic aspect of the osteochondral defect of the right medial femoral condyle, **b** resection of the cartilage defect and surface debridement of the bone defect, **c** harvesting autologous bone autograft “around the corner” of the cartilage edge, **d** impaction of the autologous bone graft and **e** implantation of autologous chondrocytes in fibrin gel

After culturing of the chondrocytes for 6 weeks, the patients returned for a second-stage reconstruction of the osteochondral defect by combined autologous bone grafting and GACI procedure. All operations were performed under general or spinal anaesthesia, and a tourniquet was used. Prophylactic antibiotics, cefuroxime (cephalosporin, Zinacef^®^) 1,500 mg IV, were administered at the induction of anaesthesia, and a second, third and fourth dose of 750 mg at 8, 16 and 24 h postoperatively. Arthrotomy was performed via a midline incision, and the joint was approached medially or laterally depending on the location of the defect. Sclerotic bone and fibrous tissue in the bed of the defect were resected and debrided using a curette until bleeding cancelous bone and a stable cartilaginous rim were obtained (Fig. 1a, b). The defect was measured, and a template was created using a small piece of aluminium foil.

Bone harvesting for the reconstruction of the osseous defect was performed using the distal femur as source of autograft bone. The ipsilateral medial or lateral distal femur was reached using the same approach 2 cm above the articular edge “around the corner” (Fig. 1c). The template was placed on the periosteum of the distal femur condyle, and an outline 1–2 mm larger than the template was marked on the periosteum. Using an osteotome, a corticospongious bone graft was harvested en bloc in the same shape and size of the defect. Subsequently, the bone graft was impacted in the defect until its cortex levelled the subchondral plate (Fig. 1d) and perforated with superficial holes with a maximum depth and diameter of 2 mm. Thereafter, the GACI procedure was performed to create the cartilage layer over the bone graft (Fig. 1e). During application, the cultured chondrocytes were mixed with thrombin and human fibrinogen (Beriplast^®^; CSL Behring, Germany) in a 1:1 ratio. Polymerization of thrombin and fibrinogen alters the liquid cell–gel mixture into a dense fibrin structure in 3 min [10, 18, 19, 37]. The fibrin gel serves as a scaffold for the chondrocytes. After polymerization, stability of the fibrin gel is tested by repeated flexion and extension of the knee. Finally, the donor site is filled up with allogen bone graft (Netherlands Bonebank Foundation, NBF), and the synovium is sutured back in place. The arthrotomy is closed, and fascia and subcutis are sutured in layers. Postoperatively, the knee was splinted in extension, and the leg was elevated for 12 h. Rehabilitation was supported by using a continuous passive motion (CPM) device during the first 6 weeks, for 6 h a day at regular intervals. Full weight bearing was allowed 8 weeks postoperative under supervision of a physiotherapist.

## Results

Eight Patients were treated for unstable osteochondral defects of the medial femoral condyle, and 1 patient was treated for an unstable osteochondral defect of the lateral femoral condyle. All defects were staged ICRS grade III–IV. The median defect size was 7.1 cm^2^ (range 2.5–12.0), and the median depth of the lesion was 0.9 cm (range 0.8–1.2).

**Fig. 2:**
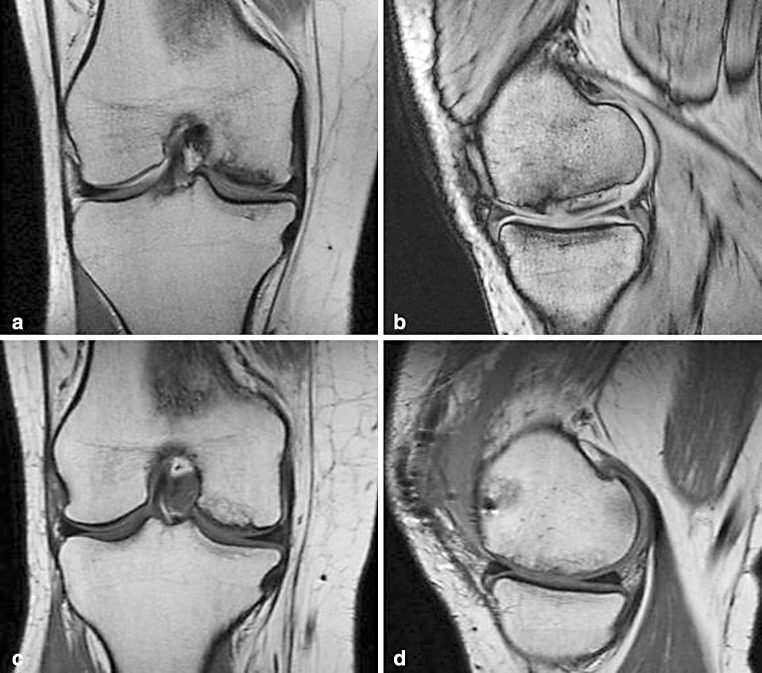
MRI images of a 31-year-old female patient treated by combined autologous bone grafting and GACI. **a** Preoperative coronal T1-weighted spin-echo MR image and **b** sagittal T1-weighted gradient echo MR image shows a severe osteochondral defect (ICRS IV) of the medial condyle in the weight-bearing zone. At 3 months postoperative follow-up, the bone repair tissue of the bone graft shows remodelling, and the signal intensity of the transplanted cartilage is comparable with the adjacent cartilage on the postoperative **c** coronal and **d** sagittal T1-weighted MR images

There were no postoperative complications. The mean IKDC score showed 6-month improvement from 35 (SD ± 16) to 51 (SD ± 18) (*n* = 9; *p* = 0.01), and a 1-year improvement from 35 (SD ± 16) to 57 (SD ± 20) (*n* = 6; *p* = 0.03). The mean KOOS score improved from 44 (SD ± 16) to 62 (SD ± 19) (*n* = 9; n.s.) at 6-month follow-up and from 44 (SD ± 16) to 65 (SD ± 24) (*n* = 6; n.s.) at 1-year follow-up. MRI at 3 months postoperative demonstrates a diminishing demarcation zone of the transplanted bone graft, and a complete filling of the cartilage defect with repair tissue to the level of the adjacent cartilage (Fig. 2). The surface of the cartilage repair tissue was intact, displaying a homogeneous structure with an iso-intense signal at T1-weighted MRI with regard to the adjacent cartilage.

One patient suffered from persistent severe complaints. MRI at 6 months showed progressive multiple cyst formation of the entire distal femur outside the treated area. The affected knee of the patient was converted to an unicompartmental arthroplasty 1 year after initial surgery. Histology of the osteochondral biopsies taken from the centre of the defect, treated by combined autologous bone grafting and GACI and showed a homogeneous modulation zone containing a mixture of fibroblasts, progenitor cells and chondroblasts. Beneath this modulation zone, chondrocytes were found. The border zone of cartilage and bone showed a reactive process of adjacent bone, fibrotic tissue and osteoblasts. There were no signs of infection or osteonecrosis.

## Discussion

The most important result to emerge from this study is that the presented surgical method makes it possible to treat severe and unstable osteochondral defects of the femoral knee by repair tissue that resembles native corticospongious bone and articular cartilage. New in our study is the combined use of autologous bone grafting and surface cartilage repair by GACI to reconstruct the osteochondral defect.

Bone grafting is necessary to fill up the osseous defect to the level of the subchondral bone plate. Usually, the proximal tibia or iliac crest has been utilized as sources for autograft bone. Unfortunately, these harvest sites are associated with substantial postoperative pain, morbidity and additional surgical exposure [3, 35]. These disadvantages can be avoided using the ipsilateral distal femur as the source of autograft bone, thereby circumventing a second surgical site; the corticospongious autograft bone can be harvested ‘around the corner’ of the cartilage edge. The autograft is harvested en block with an osteotome and subsequently size-matched by hand according to the preoperative acquired aluminium foil template. The spongious part of the graft is impacted at the internal site of the defect to facilitate bone ingrowth. If necessary, additional morselized spongious bone chips can be impacted below the corticospongious graft to level the graft surface to the joint surface. The cortical surface of the graft is placed at the outer site and replaces the original subchondral bone plate. Small superficial holes are made in the cortical surface to stimulate fixation and regeneration of the applied GACI. Since the cortical surface of the corticospongious graft originates from an anatomically slight convex cortical surface of the distal femoral condyle, there were no difficulties in using this corticospongious graft for convex subchondral surface reconstruction of the articular knee joint. In addition, some further moulding of the thin cortex in a convex surface proved to be possible.

Surface cartilage repair by ACI has been introduced for focal cartilage repair [7, 8, 21, 31, 34]. This technique encompasses culturing autologous chondrocytes for four to 6 weeks, and subsequent implantation below a periosteal patch (ACI-P) [7, 8, 31] or a porcine collagen patch (ACI-C) [21, 31] sutured and sealed with fibrin glue over the defect. Good results have been reported with these techniques for the treatment of focal cartilage defects up to four square cm [21, 28, 31, 34]. However, the treatment of osteochondral defects of the distal femur condyle by combined bone grafting and ACI-P or ACI-C has been described in only a few papers. Peterson et al. [28] described a “sandwich technique” in 7 out of 58 patients treated for symptomatic osteochondral defects of the knee. In this procedure, cancellous bone from the iliac crest or from the proximal-medial part of the tibia was harvested and covered with two periosteal membranes. Subsequently, autologous chondrocytes were injected between the two periosteal layers. Bartlett et al. [5] described a variation in this method using a bilayer porcine collagen matrix seeded with chondrocytes to cover the applied bone graft in 8 patients. All 8 patients showed a significant improvement on the Cincinatti score 6 months postoperatively, and 5 patient were available for 1-year follow-up. At 1-year follow-up, 4 out of these 5 patients demonstrated good or excellent results [5]. Ochs et al. [25] described the results of a two to 5-year follow-up of 26 patients with symptomatic osteochondral defects of the knee. The defect was filled up with monocortical cancellous cylinders, harvested from the iliac crest. The cylindrical cortical graft layers served as novel subchondral bone plate, which was subsequently covered with a chondrocyte-seeded matrix fixated with sutures. At final follow-up, 73% of all patients demonstrated good or excellent results [25]. Finally, Krishnan et al. [21] reported the 2- to 7-year result of 37 patients treated by ACI-C for symptomatic osteochondral defects of the knee. However, only one patient was treated by combined bone grafting and ACI-C.

To our best knowledge, the use of a gel-type ACI (GACI) in combination with bone grafting for severe unstable osteochondral defects of the knee has not been reported before. GACI has been introduced as an alternative ACI procedure for the repair of focal cartilage lesions of the knee [10, 18, 19, 37]. The fibrin gel fills up to the exact shape of the chondral lesion and polymerizes within 3 min after application. The use of GACI without using a periosteal or collagen patch reduces surgical difficulties associated with fixation and sealing of the patch to the intact cartilage edge. In addition, the gel facilitates the attachment and distribution of the cultured chondrocytes in the defect. Finally, a lower incidence of graft-associated complications such as fibrillation, hypertrophy or delamination has been reported in GACI when compared to ACI-P [10, 14, 18, 29, 38].

During surgery, the stability of the graft and GACI was tested by passive flexion and extension of the knee joint. If the graft and GACI adequately remains in the defect site, the surgery was finished. Immediate postoperative brace immobilization of the knee in extension is advised. In addition, early intermitted CPM has been used for rehabilitation, as recommended by Salter et al. [30]. Immediate postoperative CPM has beneficial effects on cartilage healing. In addition, it has been shown that CPM promotes postoperative wound healing following arthrotomy [33].

One failure following the treatment of a severe osteochondral defect of the knee by GACI combined with autologous bone grafting was experienced. This 39-year-old female suffered from a traumatic osteochondral defect 6 years before she entered our study. A reason for this failure may have been the development of secondary osteoarthritis of the medial femur condyle during these years. Therefore, local treatment of the osteochondral defect of this patient by GACI was not possible anymore and failed. The patient was treated with an unicompartmental knee arthroplasty 1 year after GACI treatment. Conversion to a total or unicompartmental knee arthroplasty is warranted when the clinical symptoms and additional imaging suggest treatment failure. A second-look arthroscopy in all treated patients combined with biopsy of the grafted surface would enable us to determine the histological quality of the restorative tissue, but due to ethical considerations, this was not performed in this patient group.

Limitations of this study are the small number of patients and short follow-up time. However, the incidence of patients with severe symptomatic and unstable osteochondral defects of the knee is (fortunately) low. Therefore, a larger prospective controlled study is necessary to evaluate the advantages of this technique compared to combined autologous bone grafting and ACI-C or ACI-P as a treatment option for severe and unstable osteochondral defects of the knee.

## Conclusion

Combined autologous bone grafting and a gel-type ACI (GACI) may offer an alternative and technically less demanding surgical option for severe and unstable osteochondral defects of the knee.

## Abbreviations

ACI: Autologous chondrocyte implantation
ACI-P: Autologous chondrocyte implantation using periosteal membranes
ACI-C: Autologous chondrocyte implantation using collagen membranes
GACI: Gel-type autologous chondrocyte implantation
IKDC: International knee documentation committee
KOOS: Knee injury and osteoarthritis outcome score
MRI: Magnetic resonance imaging
ICRS: International cartilage repair society
CPM: Continuous passive motion
